# Chemical Characterization of Sauvignon Blanc Wines from Three Cold-Climate-Growing Areas of Chile

**DOI:** 10.3390/foods13131991

**Published:** 2024-06-24

**Authors:** Javiera Rojas, Claudia Viacava, Cristina Ubeda, Álvaro Peña-Neira, Italo F. Cuneo, Nathalie Kuhn, Alejandro Cáceres-Mella

**Affiliations:** 1 Facultad de Ciencias Agronómicas y de los Alimentos, Pontificia Universidad Católica de Valparaíso, Av. San Francisco s/n, Quillota 2260000, Chile; javiera.rojas.a@mail.pucv.cl (J.R.); claudia.viacava.g@mail.pucv.cl (C.V.); italo.cuneo@pucv.cl (I.F.C.); nathalie.kuhn@pucv.cl (N.K.); 2 Departamento de Nutrición y Bromatología, Toxicología y Medicina Legal, Facultad de Farmacia, Universidad de Sevilla, C/Profesor García González 2, 41012 Sevilla, Spain; c_ubeda@us.es; 3 Facultad de Ciencias Agronómicas, Universidad de Chile, Av. Santa Rosa 11315, Santiago 8820808, Chile; apena@uchile.cl

**Keywords:** Sauvignon Blanc, phenolic compounds, wines, aromatic compounds, geographical area

## Abstract

The influence of the geographical location on the chemical composition of commercial Sauvignon Blanc wines was investigated. The assay was carried out on Sauvignon Blanc wines from three cold-climate valleys in Central Chile, Casablanca, Leyda, and San Antonio. The analyses revealed clear variations in some chemical parameters, especially in titratable acidity, which was higher in the geographical areas closest to the Pacific Ocean, such as the Leyda and San Antonio valleys. Regarding the composition of low-molecular-weight phenolic compounds, 17 compounds were found, and the results show that the Casablanca valley exhibits a greater abundance of monomeric flavanols, such as (+)-catechin, whereas the Leyda valley shows a higher abundance in flavonols and phenolic acids esterified with tartaric acid. Concerning the aromatic compound profile, the wines from the Casablanca valley showed a greater abundance of esters, C_13_ norisoprenoids, and some terpenes. The PLS-DA analysis revealed some differences, especially between wines from Casablanca and Leyda, demonstrating that the difference in the chemical composition of the wines was influenced by the geographical area.

## 1. Introduction

The characteristics of a wine are intimately linked to the soil and climatic conditions of the valley or area where it is cultivated; this is known as terroir, and it is something that has been studied in different parts of the world [1]. The Sauvignon Blanc variety is one of the main white varieties in the world in terms of its surface area and volume of wine, and it is cultivated in various climate and soil conditions. For this reason, it is possible to find various types of Sauvignon Blanc depending on the region of origin, with some being very famous, especially those from cold climates, such as those produced in New Zealand and France. Conversely, there are other types of Sauvignon Blanc produced in areas considered as warmer, such as Australia, the USA, and Chile [2]. For approximately 30 years, new cold climate areas have been developed in Chile for Sauvignon Blanc production, such as the Casablanca, Leyda, and San Antonio valleys. Currently, in Chile, the surface area of this variety is 14,456 hectares (11.2% of the national total), with 2998 hectares concentrated in the Casablanca, Leyda, and San Antonio valleys [3].

The grape of this variety exhibits a medium-to-high sugar content and is balanced in total acidity and pH. In the wines, the perceived aromas range from more vegetal notes, such as green pepper, boxwood, and cat urine, to fruitier aromas, like grapefruit and passion fruit. These aromas are attributed to the presence of aromatic compounds such as methoxypyrazines, such as 3-isobutyl-2-methoxypyrazine (IBMP) with green pepper notes and 3-isopropyl-2-methoxypyrazine (IPMP) with more vegetal notes [4], and volatile thiols including 3-sulfanylhexan-1-ol (3SH) with grapefruit aromas, 3-sulfanylhexyl acetate (3SHA) with passion fruit aromas, and 4-methyl-4-sulfanylpentan-2-one (4MSP) with boxwood aromas [5,6,7]. Because the climate can influence the aromatic composition of grapes and consequently of wines, some wines are considered more vegetal (green pepper, asparagus, boxwood, and tomato leaf), while others are more tropical (gooseberry, grapefruit, and passion fruit) [8].

In addition to these compounds, other classes, such as C_13_-norisoprenoids, terpenes, and other compounds, derived from the aromas produced during the wine fermentation stage, such as esters, are also crucial for the fruity aromas of white wines like Sauvignon Blanc [9]. Ubeda et al. [10] investigated the aromatic profile of 2017 vintage Sauvignon Blanc wines from a warm region in the central zone of Chile (Curicó) after being stored for 6 months in different types of containers, identifying 45 volatile compounds, including 21 esters, 5 alcohols, 3 acids, 8 terpenes, 5 C_13_-norisoprenoids, and 2 volatile thiols. On the other hand, Gil i Cortiella et al. [11] investigated the volatile component of Sauvignon Blanc wines produced by the vinification of grapes from the Leyda region, comparing the different types of fermentation vessels. Using solid-phase microextraction and gas chromatography-mass spectrometry (SPME-GC–MS), they identified 45 volatile compounds, 25 esters, 8 alcohols, 7 terpenes (including 5 monoterpenes and 2 sesquiterpenes), 3 acids, 1 aldehyde, and 1 non-megastigmane (C_13_-norisoprenoid), thus demonstrating differences among wines of the same variety and country.

Conversely, phenolic compounds are significant because they impart certain characteristics to wines, affecting, for example, the bitterness and astringency of the wines, and because they are substrates that can easily oxidize, causing the wine to lose its aroma characteristics and leading to browning. Additionally, they can affect the perception threshold of certain key aromatic compounds in wines [12,13]. Gil i Cortiella et al. [11], using high-performance liquid chromatography coupled with a photodiode array detector and solid-phase extraction (HPLC-DAD-SPE) on non-commercial Sauvignon Blanc wines made with grapes from the Leyda region, detected 10 compounds (hydroxycinnamic acids and their derivatives). On the other hand, Olejar et al. [14], in Sauvignon Blanc wines from New Zealand, determined using HPLC-DAD 12 low-molecular-weight phenolic compounds, gallic, syringic, caffeic, caftaric, *p*-coumaric, coutaric, ferulic acids, the flavan-3-ol monomers (+)-catechin and (–)-epicatechin, stilbene resveratrol, the oxidation product GRP, and finally quercetin-3-*O*-glucoside.

The viticultural valleys of Casablanca, Leyda, and San Antonio lie between the Pacific Ocean and the Coastal Range, resulting in a pronounced coastal influence. For example, in a study to determine the *terroir* effect, it was observed that, in the Maule Valley, vines growing in sites closer to the Pacific Ocean exhibit higher concentrations of amino acids and volatile compounds in grapes and wines, whereas vines growing in sites further away from the sea produce grapes that yield wines with higher alcohol and phenolic concentrations [15]. Several studies have explored the geographical classification of Sauvignon Blanc wines based on their chemical and sensory characteristics [16,17,18,19,20,21]. However, to our knowledge, there is limited literature examining the same under the coastal geographical conditions of central Chile, which could serve as an initial step towards differentiating wines based on their geographical origin. Therefore, the aim of this research was to chemically characterize, particularly focusing on low-molecular-weight phenols and aromatic compounds, on commercial Sauvignon Blanc wines from three cold-climate growing areas in central Chile.

## 2. Materials and Methods

### 2.1. Reagents and Equipment

In this study, the following reagents were used: Gallic acid, caftaric acid, (+)-catechin, quercetin, myricetin, and kaempferol standard were bought from Sigma-Aldrich (St. Louis, MO, USA). Phenolphthalein, sodium hydroxide, chlorohydric acid, starch, iodine, copper sulfate, anhydrous glucose, diethyl ether, ethyl acetate, methanol, acetic acid, acetonitrile, sodium chloride, PVDF membranes, and 4-methyl-2-pentanol were purchased from Merck (Darmstadt, Germany). Helium gas was supplied by Indura S.A (Santiago, Chile). The absorbance values were conducted using a UV-Visible spectrophotometer model UV-1280 (Shimadzu, Kyoto, Japan). The low-molecular-weight phenolic compound profile was performed in a HPLC system, Agilent 1100 Series, consisting of a G1315B diode array detector (DAD), a G1311A quaternary pump, a G1379A degasser, and a G1329A autosampler (Agilent Technologies, Santa Clara, CA, USA). A Nova-Pak C_18_ reverse phase column (4 µm, 3.9 mm I.D. × 300 mm, Waters) was used for the analysis of individual phenolic compounds. Gas chromatography analysis was carried out using a 7890B Agilent GC system coupled to an Agilent 5977 quadrupole mass spectrometer (Agilent Technologies, Santa Clara, CA, USA). The headspace solid-phase microextraction was conducted using a 2 cm 50/30 µm Caboxen/DVB/PDMS/SPME (Agilent Technologies, Santa Clara, CA, USA).

### 2.2. Wine Samples

The study was conducted on 20 commercial Sauvignon Blanc wines, all from the year 2020, and sourced from three coastal viticultural valleys in central Chile: 8 commercial wines from the Designation of Origin (D.O.) Casablanca, 7 wines from the D.O. Leyda, and 5 wines from the D.O San Antonio. The samples were collected by purchasing them from specialized stores, and several wineries included in the study also contributed with wines. Although the wines are commercial samples, there are some common practices in their production area that carried out by the local wineries and consulted by the winemakers. The grapes are harvested at good maturity and in good sanitary conditions. The pressing uses the first fractions of the must to avoid an excessive extraction of phenolic compounds. The alcoholic fermentation was carried out at a temperature of 14–16 °C, with commercial yeasts for white wine production and supplemented with amino acid and ammonium-based preparations at the doses recommended by manufacturers. The wines did not undergo malolactic fermentation. Throughout the production process, inert gases (e.g., carbon dioxide) was used to avoid excessive oxidation. The commercial prices of the wines ranged between USD 7 and USD 15 at the point of sale (retail price). Three bottles were used for each type of wine, corresponding to three replicates. Table 1 shows some climatic parameters of the three zones under study during the January to March season of 2020.

### 2.3. Global Chemical Analyses and Spectrophotometric Characterization of Wine Samples

The analyses of volatile acidity (g acetic acid L^−1^), titratable acidity (g tartaric acid L^−1^), residual sugar (g glucose L^−1^), alcohol content (% *v*/*v*), and pH were carried out using analytical methods recommended by the International Organisation of Vine and Wine (OIV) (OIV, 2012) [22]. The measure of total phenols was conducted at 280 nm by using a UV spectrophotometry, with gallic acid as the standard following the methodology of Glories [23]. The color intensity of the wines was obtained through spectrophotometry by measuring the absorbance at 420 nm with a 10 mm optical path cuvette [23].

### 2.4. HPLC-DAD Analyses of Low-Molecular-Weight Phenolic Compounds in Wine Samples

The low-molecular-weight phenolic compounds were examined using high-performance liquid chromatography coupled with a diode array detector (HPLC-DAD). Phenolic compounds were obtained from a 50 mL wine sample using liquid–liquid extractions with diethyl ether (3 × 20 mL) and ethyl acetate (3 × 20 mL). The resulting extract was evaporated to dryness at 30 °C and dissolved in 2 mL of a methanol:water solution (1:1 *v*/*v*), and then filtered using a 0.22 µm PVDF membrane. An aliquot of 25 µL was subjected to chromatographic separation at 20 °C. The DAD detector was set between 210 and 360 nm. Two mobile phases were used: A, water:acetic acid (98:2 *v*/*v*), and B, water:acetonitrile:acetic acid (78:20:2 *v*/*v*). The gradient was applied at a flow rate of 1 mL min^−1^ from 0 to 55 min and 1.2 mL min^−1^ from 55 to 90 min: 100–20% A, 20–10% A from 55 to 57 min, and 10–0% A from 57 to 90 min. Each peak in the chromatogram was characterized in terms of its retention time and absorbance spectrum. Quantification was performed using the external standard method with commercial standards. The calibration curves used for the quantification of each compound were prepared under the same conditions as the samples [24].

### 2.5. SPME-GC-MS Analyses for Volatile Compounds in Wine Samples

The analysis of the aromatic composition of the wines was conducted using the methodology described by Ubeda et al. [25]. The solid-phase microextraction (SPME) technique was employed for the extraction of the volatile compounds, where 7.5 mL of wine was placed in a 20 mL glass vial containing 1.5 g of sodium chloride and 10 µL of 4-methyl-2-pentanol, added as an internal standard. The samples were incubated at 45 °C with agitation (500 rpm) for 20 min. After this time, the SPME fiber was exposed to the headspace of the vial for 40 min to adsorb the odorous molecules. Following this, the fiber had a desorption tine over 180 s. The sample was injected using the splitless mode with a transfer line temperature of 280 °C. The identification of compounds on the fiber was performed using an Agilent 7890B chromatographic system (Agilent Technologies, Santa Clara, CA, USA). The chromatographic system had a DB Wax capillary column with dimensions of 60 × 0.25 mm and a thickness of 0.25 µm using a helium flow rate of 1 mL min^−1^. The oven temperature program was as follows: 35 °C for 1 min, increased to 130 °C at 12 °C/min and held for 1 min, then increased to 160 °C at 1 °C/min and subsequently reaching 220 °C at 10 °C/min, maintaining for 10 min. Compound detection was carried out using an Agilent 5977 mass spectrometer (Agilent Technologies, Santa Clara, CA, USA) with electron ionization in the scanning mode registered at 70 eV in the range of 35 to 300 u. ChemStation software (B03.02 version) was used for chromatogram analysis. Compound identification was performed by comparing spectra with the NIST database (Version 2.0) and the linear retention index (LRI) of authentic standards, calculated from the retention times of n-alkanes under identical conditions for each analysis program.

### 2.6. Statistical Analyses

The statistical analyses were conducted using the statistical software R, version 4.2.1. For the analysis of the chemical data, an analysis of variance (ANOVA) and least significant difference test (LSD) were performed with a significance level of 95% (*p* < 0.05). Additionally, a partial least squares discriminant analysis (PLS-DA) was carried out to identify metabolomic data in the wine samples using The Unscrambler^®^ X version 10.4 software (Camo, Oslo, Norway).

## 3. Results

### Global Chemical Composition in Sauvignon Blanc Wine Samples

In Table 2, the concentration of basic parameters in the evaluated wines can be observed. In the case of total phenols, the concentrations ranged from 116.59 to 139.70 mg GAE L^−1^ with no differences between valleys. The color of the wines ranged from 0.14 to 0.07 with no differences between valleys. Regarding alcohol content, it ranged from 13.46 to 13.78% *v*/*v*. Meanwhile, the concentration of reducing sugars ranged from 0.64 to 1.24 g glucose L^−1^.

The volatile acidity showed similar values among wines from different valleys, ranging from 0.28 to 0.40 g acetic acid/L. As for the pH of the wines, it ranged from 2.83 to 2.91. No differences between valleys were observed in the above parameters. Finally, the concentration of the titratable acidity ranged from 5.71 to 6.66 g tartaric acid L^−1^, and it was observed that wines from the Leyda and San Antonio valleys exhibited a higher concentration of acids compared to wines from the Casablanca area.

**Figure 1:**
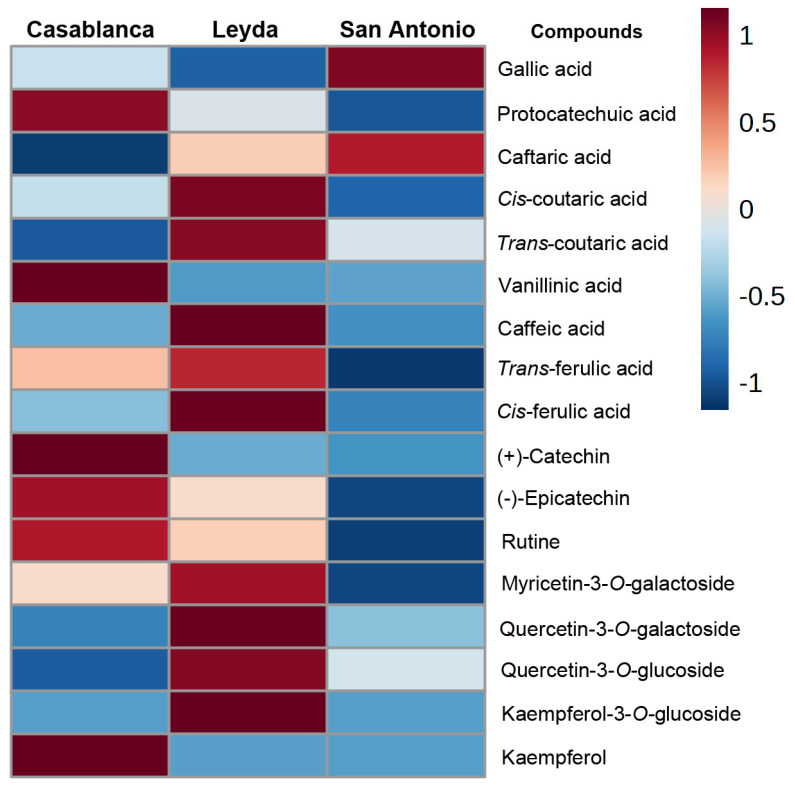
Heatmap of low-molecular-weight phenolic compounds identified by HPLC-DAD in commercial Sauvignon Blanc wines from three cold-climate valleys.

Figure 1 shows a heatmap displaying the abundance of phenolic compounds measured in commercial Sauvignon Blanc wines from the three cold-climate valleys.

The results show the presence of 17 phenolic compounds, including 9 phenolic acids, 2 compounds belonging to the monomeric flavanols family, and 6 compounds belonging to the flavonols (Table S1). The wines from Casablanca exhibited a relative abundance in protocatechuic acid, vanillic acid, (+)-catechin, (-)-epicatechin, and the flavonols rutin and kaempferol. Regarding the wines from the Leyda area, they displayed a relative abundance in *cis*- and *trans*- *p*-coutaric acids, caffeic acid, *trans*- and *cis*- ferulic acids, and the flavonols myricetin-3-*O*-galactoside, quercetin-3-*O*-galactoside, quercetin-3-*O*-glucoside, and kaempferol-3-*O*-glucoside. On the other hand, the San Antonio wines only exhibited a relative abundance in gallic acid and caftaric acid. In general terms, the wines from Casablanca exhibited a higher concentration of the flavanol (+)-catechin, whereas the Leyda wines showed a greater abundance in flavonols and phenolic acids esterified with tartaric acid, such as *trans*- and *cis*-coutaric acids and ferulic acids, in both forms. Finally, the wines from the San Antonio area displayed a lower relative abundance of low-molecular-weight phenols compared to the other regions and no presence of monomeric flavanols.

**Figure 2:**
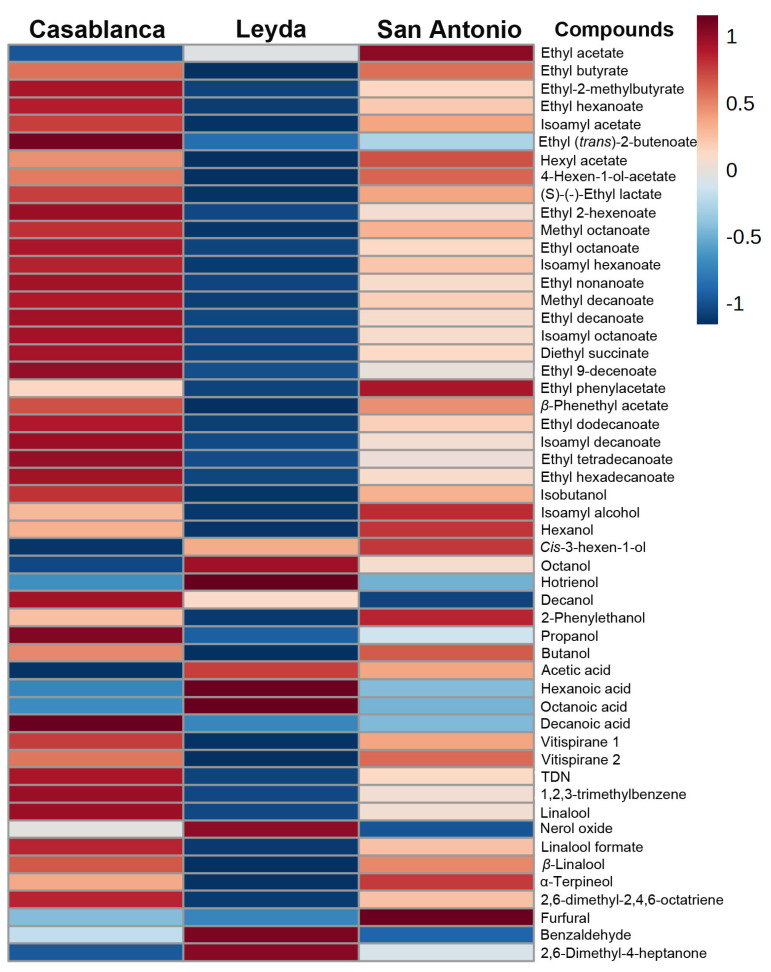
Heatmap of the relative area of aromatic compounds measured by GC-MS in commercial Sauvignon Blanc wines from three cold-climate valleys.

Figure 2 shows the heatmap displaying the concentration of aromatic compounds found in commercial Sauvignon Blanc wines from the three cold-climate valleys.

A total of 52 aromatic compounds were found, of which 25 compounds belong to the ester family, 10 compounds are alcohols, 4 compounds belong to the acid category, 4 compounds are part of the C_13_-norisoprenoid family, 6 compounds are terpenes, 2 are aldehydes, and 1 is a ketonic compound. The wines from San Antonio exhibited a relative abundance in ethyl acetate, ethyl butyrate, hexyl acetate, 4-hexen-1-ol acetate, ethyl phenylacetate, β-phenethyl acetate, isoamyl alcohol, hexanol, cis-3-hexen-1-ol, 2-phenylethanol, butanol, vitispirane 2, α-terpineol, and furfural. On the other hand, the wines from the Leyda area showed a relative abundance in octanol, hotrienol, acetic acid, hexanoic acid, octanoic acid, nerol oxide, benzaldehyde, and 2,6-dimethyl-4-heptanone. Lastly, the wines from the Casablanca area exhibited a relative abundance in the remaining identified aromatic compounds, especially in the majority of esters, C_13_-norisoprenoids, and terpenes. In general, among the esters group, the wines from Casablanca displayed a higher relative abundance, whereas this was much lower in Leyda.

**Figure 3:**
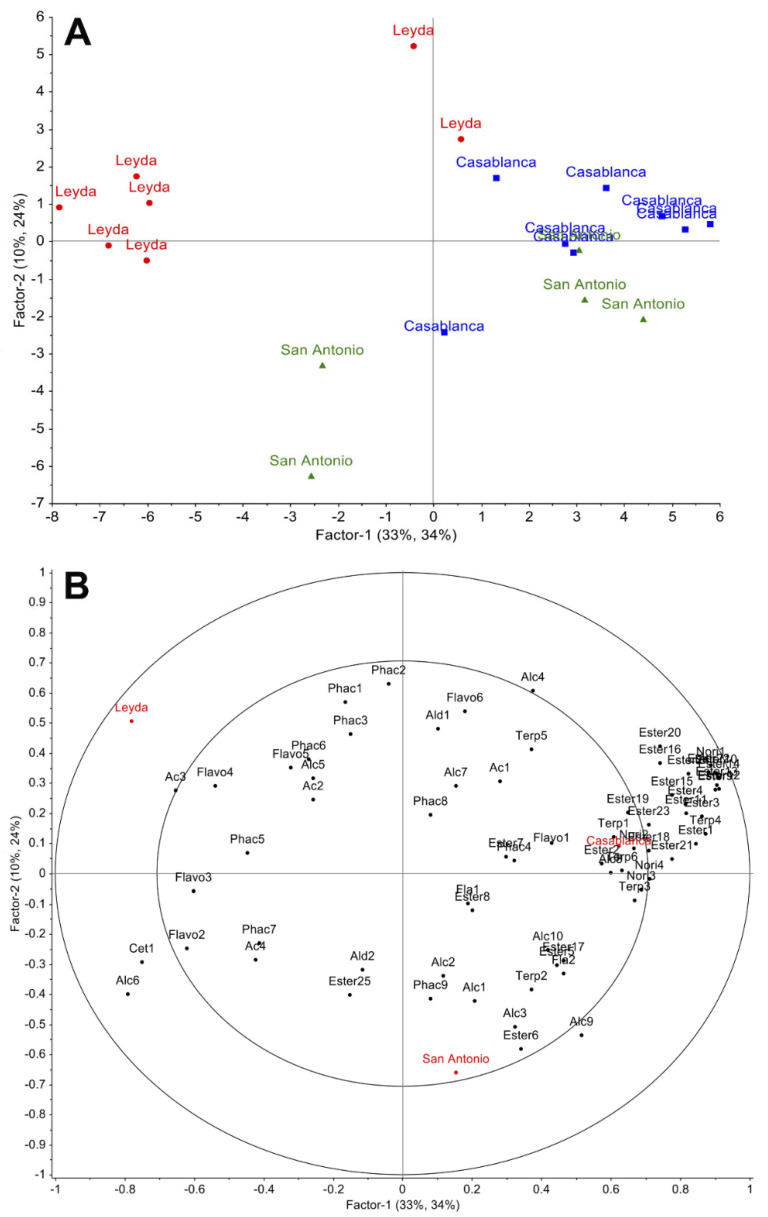
PLS-DA analysis based on the chemical composition of low-molecular-weight phenols and aromatic compounds measured in commercial Sauvignon Blanc wines from three cold-climate valleys. (**A**): scores and (**B**): loadings. Phenolic compounds: Flavo1, kaempferol; Flavo2, kaempferol-3-*O*-glucoside; Flavo3, quercetin-3-*O*-glucoside; Flavo4, quercetin-3-*O*-galactoside; Flavo5, myricetin-3-*O*-galactoside; Flavo6, rutine; Fla1, (−)-epicatechin; Fla2, (+)-catechin; Phac1, *cis*-ferulic acid; Phac2, *trans*-ferulic acid; Phac3, caffeic acid; Phac4, vanillinic acid; Phac5, *trans*-coutaric acid; Phac6, *cis*-coutaric acid; Phac7, caftaric acid; Phac8, protocatechuic acid; Phac9, gallic acid. Aromatic compounds: Cet1: 2,6-dimethyl-4-heptanone; Ald1, benzaldehyde; Ald2, furfural; Terp1, 2,6-dimethyl-2,4,6-octatriene; Terp2, α-terpineol; Terp3, β-linalool; Terp4, linalool formate; Terp5, nerol oxide; Terp6, linalool; Nori1, 1,2,3-trimethylbenzene; Nori2, TDN; Nori3, Vitispirane 2; Nori4, Vitispirane 1; Ac1, decanoic acid; Ac2, octanoic acid; Ac3, hexanoic acid; Ac4, acetic acid; Alc1, butanol; Alc2, propanol; Alc3, 2-phenylethanol; Alc4, decanol; Alc5, hotrienol; Alc6, octanol; Alc7, cis-3-hexen-1-ol; Alc8, hexanol; Alc9, isoamyl alcohol; Alc10, isobutanol; Ester1, ethyl hexadecanoate; Ester2, ethyl tetradecanoate; Ester3, isoamyl decanoate; Ester4, ethyl dodecanoate; Ester5, β-phenethyl acetate; Ester6, ethyl phenyl acetate; Ester7, ethyl 9-decenoate; Ester8, diethyl succinate; Ester9, isoamyl octanoate; Ester10, ethyl decanoate; Ester11, methyl decanoate; Ester12, ethyl nonanoate; Ester13, isoamyl hexanoate; Ester14, ethyl octanoate; Ester15, methyl octanoate; Ester16, ethyl 2-hexenoate; Ester17, (S)-(-)-ethyl lactate; Ester18, 4-hexen-1-ol-acetate; Ester19, hexyl acetate; Ester20, ethyl (trans)-2-butenoate; Ester21, isoamyl acetate; Ester22, ethyl hexanoate; Ester23, ethyl-2-methylbutyrate; Ester24, ethyl butyrate; Ester25, ethyl acetate.

Figure 3 shows the partial least square discriminant analysis (PLS-DA) using chemical data on the composition of low-molecular-weight phenols and aromatic compounds. A total of 69 chemical variables were employed, comprising 17 phenolic compounds and 52 aromatic compounds, using two factors. Factor 1 represented 33% of the variability, while factor 2 explained 10% of the variability. Together, both components accounted for 43% of the total variance. The results indicate that only certain wines were differentiated between the valleys, particularly demonstrating a clear distinction between the wines from Casablanca and the Leyda area. It is evident that the samples from Leyda and San Antonio are further away from their centroid, implying a significant dispersion among the analyzed samples for the variables defining this multivariate analysis.

## 4. Discussion

The research on the chemical composition of wines and how various variables localized in geographical areas can impact certain chemical and sensory characteristics of these wines is becoming increasingly important, especially in the context of a changing climate [2]. Many consumers prefer certain wine characteristics that may be influenced by the features of a particular geographical area. The results indicate that wines from the Leyda and San Antonio areas exhibited a higher titratable acidity compared to wines from Casablanca, although there were no differences in terms of pH among them. This could be attributed to climatic differences between the valleys, as San Antonio and Leyda are closer to the Pacific Ocean, where coastal influence may have led to higher acidity in the wines due to reduced organic acid respiration in the berries, given the lower temperatures during grape ripening. In previous studies, in the Casablanca area, the sub-area closest to the Pacific Ocean showed a higher acidity [26], which could be comparable to the two valleys much closer to the Pacific Ocean. Additionally, it is important to note that commercially produced wines may have defined styles by each winery, which can impact these parameters, as vinification methods affect the chemical composition of wines [27]. However, for white wines, it is very important and desirable to have higher total acidity [28], making this particularly important for wines from Leyda and San Antonio.

Although the relationship between pH and titratable acidity is inversely proportional, in oenology, this relationship is not as straightforward, as evidenced by the absence of differences in the pH of the wines. This may be due to the presence of mineral elements such as calcium and potassium, which can cause differences in titratable acidity but not in pH [29]. Since the presence of these compounds is desirable in white wines to impart acidity and freshness, the concentration of these elements could be interesting to study in future research. While basic parameters may be of interest to study, there are other chemical compounds that could allow for greater differentiation among wines, and these could be phenolic compounds and aromatic compounds.

In the analysis of phenolic compounds in wines, clear differences were observed among different geographical regions. Casablanca exhibited a higher proportion of monomeric flavanols, especially (+)-catechin, unlike wines from Leyda, which showed a lower concentration, and these compounds were not detected in wines from San Antonio. Conversely, wines from the Leyda area displayed a higher concentration of flavonols and phenolic acids bound to tartaric acid, such as caftaric acid, which was the phenolic compound with the highest individual concentration in this area and in San Antonio, consistent with previous studies [30,31]. In contrast, wines from the San Antonio area showed a lower concentration of phenolic compounds overall, although a high concentration of gallic acid was observed. Phenolic compounds in white wines are important as substrates for future browning reactions that may occur in wines [12,13], and their presence is not always desired by winemakers. For example, (−)-epicatechin and caftaric acid are major contributors to browning in wines as both compounds participate in the production of quinones due to the action of the enzyme polyphenol oxidase in the presence of oxygen [32]. Accordingly, both the Leyda and Casablanca regions would present a higher concentration of these compounds, making the wines potentially more prone to browning issues, affecting their sensory quality. Additionally, the high concentration of monomeric flavanols in Casablanca wines could lead to bitterness problems in these wines [30], although this should be confirmed with sensory analysis during wine aging.

In addition to phenolic compounds, the aromatic composition of wines is a tool that allows for their differentiation [33]. Chemical analysis revealed interesting results, where wines from the Casablanca area exhibited more esters than those from San Antonio and significantly more than those from Leyda. Esters in wine evoke fruity aromas such as apple, banana, honey, pear, some floral notes, and creamy aromas, specially produced during the wine fermentation stage [34]. Furthermore, wines from the Casablanca area showed a higher proportion of C_13_-norisoprenoid compounds like Vitispirane 1, which has sensory descriptors of floral and eucalyptus aromas, and TDN and 1,2,3-trimethylbenzene, which have sensory descriptors of kerosene and petroleum aromas, being very common in Riesling wines [35]. In previous studies, higher concentrations of TDN were found in Riesling wines from warmer climates [36], which could be explained in this study by the Casablanca area experiencing higher temperatures than the other regions (Table 1). In the Casablanca area, there are also aromas of tropical and citrus fruits due to the presence of 2,6-dimethyl-2,4,6-octatriene and some terpenes such as β-linalool and linalool formate [37].

In the Leyda area, wines also displayed floral and fruity aromas, although the presence of certain compounds such as 2,6-dimethyl-4-heptanone, with notes of pineapple and mint, and some derived terpenes, like nerol oxide with hints of orange blossom, and hotrienol, with citrus and linden flower notes [34], would differentiate these wines from those of Casablanca. The same trend is observed in wines from the San Antonio area. Although they also exhibit aromas that are present in the other regions with fruity and floral notes, there are other compounds that contribute to an aromatic profile distinct from the rest of the regions. For instance, these wines displayed a relative abundance of compounds such as α-terpineol with lilac flower aromas, 2-phenylethanol with floral rose aromas, *cis*-3-hexen-1-ol with freshly cut grass aromas, and 4-hexen-1-ol acetate with green aromas [38]. In this case, these aromas would be classified as more vegetal compared to those of the Casablanca and Leyda areas, which exhibited more fruity aromas, indicating that there is differentiation among the regions based on the types of aromatic compounds present. This is consistent with previous studies as Sauvignon Blanc wines produced in warmer regions express more tropical fruit aromas and fewer vegetal aromas [2]. Similarly, previous research has demonstrated that higher temperatures lead to a lower level of aromatic expression in white varieties, while cooler conditions preserve the aromas of white cultivars, such as Gewurztraminer, Sauvignon Blanc, or Riesling [39,40,41,42].

In previous studies, the differentiation of wines according to their origin has also been confirmed [27], where other aromatic compounds from the group of volatile thiols and methoxypyrazines, which are also found in the Sauvignon Blanc variety and belong to its primary aromas [27,38], appear. In a future study, quantifying these specific compounds, which are important for the sensory notes of Sauvignon Blanc wine, could shed more light on the differentiation of wines by regions, leading to an in-depth classification based on the chemical composition of the wines.

The differences among the wines found in this study in terms of their chemical composition could be attributed to various factors. As commercial wines, each winery may employ different winemaking styles, but an important aspect of Sauvignon Blanc wine is its pale yellow color, intense aromatic concentration, and high acidity [6,26]. Therefore, the differentiation that may occur among each wine is reduced, as achieving the desired characteristics in these wines requires a good harvest maturity with a high concentration of acids and the use of antioxidant compounds along with reducing the contact time between the must and the solid parts of the berry, thereby avoiding the excessive extraction of phenolic compounds, resulting in the characteristic pale yellow hues of these wines. Another factor is climatic, where it has been described that differences in these variables can influence the accumulation of chemical compounds in the berries that will later be transferred to the wine [43]. Much of the research conducted on wines from various geographical areas [1,2] considers the climatic conditions of a particular place for differentiation. In other words, the differences found in some climatic variables of the studied areas (Table 1) could explain the chemical differences in the wines. Colder areas allow for a greater accumulation of organic acids during berry formation and less respiration of these acids during ripening, which explains the higher concentration in the Leyda and San Antonio areas closer to the Pacific Ocean, where maritime influence allows for cooler conditions, ideal for the greater accumulation of acids in the wines. These differences in climatic variables could also explain the phenolic and aroma composition in the wines. Phenolic compounds, originating from the secondary metabolism of plants, depend on light and temperature for their accumulation [44], and the differences found in these parameters could explain the accumulation of some compounds. It is notably observed that the San Antonio area exhibited a lower concentration of phenolic compounds, which could be explained by San Antonio being closer to the Pacific Ocean, with cooler climatic conditions than the other regions, particularly different from the Casablanca area, which is further from the sea and also due to the lower solar radiation observed in the San Antonio area (Table 1), which could explain the overall lower concentration of phenolic compounds observed in the wines from this area. Similarly, slower ripening could be associated with less cell wall degradation in the berries and, therefore, less release of phenolic compounds during pressing.

As a means of integrating the previous results and understanding how chemical variables could differentiate wines from different regions, the use of multivariate tools could allow for the differentiation of geographical areas based on the chemical composition of the wines. In our results, the analysis was able to corroborate the findings in Figure 1 and Figure 2, where it was demonstrated that there was differentiation between regions, especially between the Casablanca and Leyda areas, whereas some wines from the San Antonio area were also related to some wines from the Casablanca area. It is worth noting that the observation of the wines used from the Casablanca area allowed them to be more clustered than the wines from San Antonio and Leyda. This could mean that the wines exhibited high variability in their composition, especially those from these two valleys, more so than the wines from the Casablanca area, or that the winemaking techniques used in these areas allow for more differentiated wines. Lastly, the soil and climatic variables in these areas could cause these differences, which raises the need for an in-depth analysis that takes into consideration other chemical compounds, especially aromatic compounds from the group of methoxypyrazines and volatile thiols, which would help to translate the differences found and associate them with the sensory typicality of this variety. A similar result, albeit in Sauvignon Blanc grapes, was proposed by Peirano-Bolelli et al. [26] in the Casablanca area, where two sub-areas were found within a broader geographical zone, thus allowing for a greater differentiation of wines in terms of their geographic origin.

## 5. Conclusions

The results of this research demonstrated that there may be differentiation in the chemical composition of wines originating from different geographical areas. Sauvignon Blanc wines from Leyda and San Antonio showed a higher acidity. About the phenolic composition, the wines from Casablanca exhibited more monomeric flavanols and Leyda presented a higher abundance of flavonols, and phenolic acid esterified with tartaric acid, such as coutaric and ferulic acids; instead, San Antonio showed the lowest content of phenolic compounds. The aromatic composition showed interesting results with the wines from Casablanca showing a higher abundance of esters, C_13_-norisoprenoids, and terpenes with citric and tropical notes. On the other hand, the wines from Leyda presented more floral aromas with orange blossom and linden flowers notes; instead, the San Antonio wines exhibited aromas such as lilac flower, rose and especially green aromas, due principally to the presence of *cis*-3-hexen-1-ol with notes of freshly cut grass and 4-hexen-1-ol with green aromas, demonstrating the differentiation of the wines based in his chemical composition. In future studies, and as an expansion of the results presented in this research, a study that takes into consideration key compounds for varietal typicity in this cultivar correlated with a sensory analysis of the wines by an expert panel, as well as the detailed study of subzones within these geographical areas and repetition over two or more years, could provide even more information for a new classification into smaller subzones, allowing for quality differentiation in the wines of this variety and expanding the range of wines produced from this variety.

## Figures and Tables

**Table 1:** Climatic data from January to March in the areas of Casablanca, Leyda, and San Antonio.

	Casablanca			Leyda			San Antonio		
	January	February	March	January	February	March	January	February	March
Maximum temperature (°C)	28.1	27.9	27.3	21.4	21.7	20.8	21.7	22.0	21.4
Minimum temperature (°C)	9.7	8.0	9.0	12.3	12.0	12.0	10.7	8.4	9.4
Thermal oscillation (°C)	18.3	19.9	18.2	9.1	9.6	8.7	11.0	13.6	12.0
Relative humidity (%)	69.0	70.3	74.6	84.3	82.2	87.6	79.3	79.5	83.2
Precipitation (mm)	0.0	0.0	0.0	0.0	0.0	0.0	0.0	0.0	0.0
Solar radiation (W m^−2^)	922.2	892.0	754.9	952.1	921.3	899.4	606.4	655.3	668.0
Maximum wind speed (m s^−1^)	2.4	2.3	2.2	4.1	4.0	3.7	3.8	3.9	3.4
Day degrees (base 10 °C)	686.3	948.81	1187.2	930.3	1139.2	1334.3	432.0	604.4	769.1

Data are from the Agroclimatic system AGROMET (Red Agroclimática Nacional) of Chile.

**Table 2:** Global analytical parameters in Sauvignon Blanc wines from three cold-climate valleys.

	Casablanca (n = 8)	Leyda (n = 7)	San Antonio (n = 5)
Total phenols (mg GAE L^−1^)	125.81 ± 2.61	116.59 ± 7.52	139.7 ± 11.27
Color (A.U 420 nm)	0.08 ± 0.00	0.07 ± 0.00	0.14 ± 0.04
Volatile acidity (g acetic acid L^−1^)	0.28 ± 0.03	0.40 ± 0.02	0.34 ± 0.07
Titratable acidity (g tartaric acid L^−1^)	5.71 ± 0.14 b	6.64 ± 0.15 a	6.66 ± 0.21 a
Reducing sugars (g/L)	0.84 ± 0.20	1.24 ± 0.55	0.64 ± 0.10
Alcoholic strength (% *v*/*v*)	13.54 ± 0.19	13.46 ± 0.15	13.78 ± 0.15
pH	2.83 ± 0.04	2.91 ± 0.03	2.88 ± 0.04

Different letters in the same row denote statistically significant differences according to the LSD test (*p* < 0.05). GAE: gallic acid equivalent. A.U: absorbance units.

## Data Availability

The original contributions presented in the study are included in the article/Supplementary Materials, further inquiries can be directed to the corresponding author.

## Supplementary Materials

The following supporting information can be downloaded at: https://www.mdpi.com/article/10.3390/foods13131991/s1, Table S1: Low-molecular-weight phenolic compounds in Sauvignon Blanc wines from three cold-climate growing areas of Chile.

## References

[1] Van Leeuwen C., Friant P., Choné X., Trégoat O., Koundouras S., Dubourdieu D. (2004). Influence of climate, soil, and cultivar on terroir. Am. J. Enol. Vitic..

[2] Van Leeuwen C., Barbe J.C., Darriet P., Geffroy O., Gomès E., Guillaumie S., Helwi P., Laboyrie J., Lytra G., Le Menn N. (2020). Recent advancements in understanding the terroir effect on aromas in grapes and wines. OENO One.

[3] SAG (Setvicio Agricola y Ganadero) (2022). Catastro Vitícola Nacional. https://www.sag.gob.cl.

[4] Allen M., Lacey M., Harris R., Brown W. (1991). Contribution of methoxypyrazines to Sauvignon blanc wine aroma. Am. J. Enol. Vitic..

[5] Tominaga T., Peyrot des Gachons C., Dubourdieu D. (1998). A new type of flavor precursors in Vitis vinifera L cv. Sauvignon blanc: S-cysteine conjugates. J. Agric. Food Chem..

[6] Lund C., Thompson M., Benkwitz F., Wohler M., Triggs C., Gardner R., Heymann H., Nicolau L. (2009). New Zealand Sauvignon Blanc distinct flavour characteristics: Sensory, chemical, and consumer aspects. Am. J. Enol. Vitic..

[7] Roland A., Schneider R., Charrier F., Cavelier F., Rossignol M., Razungles A. (2011). Distribution of varietal thiol precursors in the skin and the pulp of Melon B. and Sauvignon Blanc grapes. Food Chem..

[8] Drappier J., Thibon C., Rabot A., Geny-Denis L. (2017). Relationship between wine composition and temperature: Impact on Bordeaux wine typicity in the context of global warming. Crit. Rev. Food Sci. Nutr..

[9] King E., Kievit R., Curtin C., Swiegers J., Pretorius I., Bastian S., Leigh Francis I. (2010). The effect of multiple yeasts co-inoculations on Sauvignon blanc wine aroma composition, sensory properties and consumer preference. Food Chem..

[10] Ubeda C., Peña-Neira A., Gil i Cortiella M. (2022). Combined effects of the vessel type and bottle closure during Chilean Sauvignon Blanc wine storage over its volatile profile. Food Res. Int..

[11] Gil i Cortiella M., Ubeda C., Covarrubias J.I., Peña-Neira A. (2020). Chemical, physical, and sensory attributes of Sauvignon blanc wine fermented in different kinds of vessels. Innov. Food Sci. Emerg. Technol..

[12] Danilewicz J. (2012). Review of oxidative processes in wine and value of reduction potentials in enology. Am. J. Enol. Vitic..

[13] Li H., Guo A., Wang H. (2008). Mechanisms of oxidative browning of wine. Food Chem..

[14] Olejar K., Fedrizzi B., Kilmartin P. (2015). Influence of harvesting technique and maceration process on aroma and phenolic attributes of Sauvignon blanc wine. Food Chem..

[15] Verdugo-Vásquez N., Orrego R., Gutiérrez-Gamboa G., Reyes M., Zurita-Silva A., Balbontín C., Gaete N., Salazar-Parra C. (2023). Climate trends and variability in the Chilean viticultural production zones during 1985–2015. OENO One.

[16] Marais J., Hunter J., Haasbroek P.D. (1999). Effect of canopy microclimate, season and region on Sauvignon Blanc grape composition and wine quality. S. Afr. J. Enol. Vitic..

[17] Berna A., Trowell S., Clifford D., Cynkar W., Cozzolino D. (2009). Geographical origin of Sauvignon Blanc wines predicted by mass spectrometry and metal oxide based electronic nose. Anal. Chim. Acta.

[18] Green J., Parr W., Breitmeyer J., Valentin D., Sherlock R. (2011). Sensory and chemical characterisation of Sauvignon blanc wine: Influence of source of origin. Food Res. Int..

[19] Cozzolino D., Cynkar W., Shah N., Smith P. (2011). Can spectroscopy geographically classify Sauvignon Blanc wines from Australia and New Zealand?. Food Chem..

[20] Parr W., Schlich P., Theobald J.C., Harsch M.J. (2013). Association of selected viniviticultural factors with sensory and chemical characteristics of New Zealand Sauvignon blanc wines. Food Res. Int..

[21] Chen L., Capone D., Nicholson E., Jeffery D. (2019). Investigation of intraregional variation, grape amino acids, and pre-fermentation freezing on varietal thiols and their precursors for *Vitis vinifera* Sauvignon blanc. Food Chem..

[22] OIV (2012). Compendium of International Methods of Wine and Must Analysis.

[23] Glories Y. (1984). La coleur des vins rouges, 2eme Partier. Mesure, origine et interpretation. Connais. Vigne Vin.

[24] Peña-Neira A., Caceres A., Pastenes C. (2007). Low molecular weight phenolic and anthocyanin composition of grape skins from cv. Syrah (*Vitis vinifera* L.) in the Maipo valley (Chile): Effect of clusters thinning and vineyard yield. Food Sci. Technol. Int..

[25] Ubeda C., Del Barrio-Galán R., Peña-Neira A., Medel-Marabolí M., Durán-Guerrero E. (2017). Location effects on the aromatic composition of monovarietal cv. Carignan wines. Am. J. Enol. Vitic..

[26] Peirano-Bolelli P., Heller-Fuenzalida F., Cuneo I.F., Peña-Neira A., Cáceres-Mella A. (2022). Changes in the composition of flavonols and organic acids during ripening for three cv. Sauvignon Blanc clones grown in a cool-climate valley. Agronomy.

[27] Parish-Virtue K., Herbst-Johnstone M., Bouda F., Fedrizzi B., Deed R.C., Kilmartin P.A. (2021). Aroma and sensory profiles of Sauvignon Blanc wines from commercially produced free run and pressed juices. Beverages.

[28] Volschenk H., Van Vuuren H., Viljoen-Bloom M. (2006). Malic acid in wine: Origin, function and metabolism during vinification. S. Afr. J. Enol. Vitic..

[29] Rogiers S., Zelmari A., Coetzee Z., Walker R., Deloire A., Tyerman S. (2017). Potassium in the grape (*Vitis vinifera* L.) berry: Transport and function. Front. Plant Sci..

[30] Makhotkina O., Kilmartin P. (2012). The phenolic composition of Sauvignon blanc juice profiled by cyclic voltammetry. Electrochim. Acta.

[31] Cáceres-Mella A., Peña-Neira Á., Parraguez J., López-Solís R., Laurie V.F., Canals J.M. (2013). Effect of inert gas and prefermentative treatment with polyvinylpolypyrrolidone on the phenolic composition of Chilean Sauvignon blanc wines. J. Sci. Food Agric..

[32] Roland A., Vialaret J., Razungles A., Rigou P., Schneider R. (2010). Evolution of S-Cysteinylated and S-Glutathionylated thiol precursors during oxidation of Melon B. and Sauvignon Blanc musts. J. Agric. Food Chem..

[33] Poggesi S., Darnal A., Ceci A.T., Longo E., Vanzo L., Mimmo T., Boselli E. (2022). Fusion of 2DGC-MS, HPLC-MS and sensory data to assist decision-making in the marketing of international monovarietal Chardonnay and Sauvignon blanc wines. Foods.

[34] Carpena M., Fraga-Corral M., Otero P., Nogueira R., Garcia-Oliveira P., Prieto M., Simal-Gandara J. (2021). Secondary aroma: Influence of wine microorganisms in their aroma profile, Review. Foods.

[35] Ziegler M., Gökc R., Bechtloff P., Winterhalter P., Schmarr H., Fischer U. (2019). Impact of matrix variables and expertise of panelists on sensory thresholds of 1,1,6-trimethyl-1,2-dihydronaphthalene known as petrol off-flavor compound in Riesling wines. Food Qual. Prefer..

[36] Kwasniewski M., Vanden Heuvel J., Pan B., Sacks G. (2010). Timing of cluster light environment manipulation during grape development affects C_13_ norisoprenoid and carotenoid concentrations in Riesling. J. Agric. Food Chem..

[37] Ruiz J., Kiene F., Belda I., Fracassetti D., Marquina D., Navascués E., Calderón F., Benito A., Rauhut D., Santos A. (2019). Effects on varietal aromas during wine making: A review of the impact of varietal aromas on the flavor of wine. Appl. Microbiol. Biotechnol..

[38] Benkwitz F., Tominaga T., Kilmartin P.A., Lund C., Wohlers M., Nicolau L. (2012). Identifying the Chemical Composition Related to the Distinct Aroma Characteristics of New Zealand Sauvignon blanc wines. Am. J. Enol. Vitic..

[39] Jackson D., Lombard P. (1993). Environmental and management practices affecting grape composition and wine quality—A review. Am. J. Enol. Vitic..

[40] Roujou de Boubée D., Van Leeuwen C., Dubourdieu D. (2000). Organoleptic impact of 2-methoxy-3-isobutylpyrazine on red Bordeaux and Loire Wines. Effect of environmental conditions on concentration in grapes during ripening. J. Agric. Food Chem..

[41] Peyrot des Gachons C., Van Leeuwen C., Tominaga T., Soyer J., Gaudillere J.-P., Dubourdieu D. (2005). Influence of water and nitrogen deficit on fruit ripening and aroma potential of *Vitis vinifera* L cv. Sauvignon Blanc in field conditions. J. Sci. Food Agric..

[42] Jones G., Goodrich G. (2008). Influence of climate variability on wine regions in the Western USA and on wine quality in the Napa Valley. Clim. Res..

[43] Martin D., Grose C., Fedrizzi B., Stuart L., Albright A., McLachlan A. (2016). Grape cluster microclimate influences the aroma composition of Sauvignon blanc wine. Food Chem..

[44] Gregan S.M., Wargent J., Liu L., Shinkle J., Hofmann R., Winefield C., Jordan B. (2012). Effects of solar ultraviolet radiation and canopy manipulation on the biochemical composition of Sauvignon Blanc grapes. Aust. J. Grape Wine Res..

