# Supplementary material for: Chemical Characterization of Sauvignon Blanc Wines from Three Cold-Climate-Growing Areas of Chile
Source: Foods. 2024 Jun 24;13(13):1991. doi: 10.3390/foods13131991 (PMC11241741; doi:10.3390/foods13131991)
Supplement: Supplementary file 1 [file foods-13-01991-s001.zip › foods-3031566-supplementary.pdf]

Supplementary material

Table S1. Low-molecular-weight phenolic compounds in Sauvignon Blanc wines from three cold-climate growing zones in Central Chile

Phenolic compounds		Casablanca	Leyda	San Antonio
Phenolic acids (mg/L)	Gallic acid	78.04 ± 12.68	64.07 ± 16.96	100.64 ± 2.59
	Protocatechuic acid	130.39 ± 34.34 a	77.28 ± 19.67 ab	35.33 ± 4.61 b
	Caftaric acid	98.89 ± 46.41 b	242.22 ± 63.69 ab	319.45 ± 65.14 a
	Cis-coutaric acid	91.09 ± 17.81	119.46 ± 11.90	74.79 ± 9.22
	Trans-coutaric acid	80.69 ± 15.15	154.25 ± 35.86	112.21 ± 28.02
	Vanillinic acid	58.35 ± 25.78	24.28 ± 2.99	25.06 ± 1.54
	Caffeic acid	30.04 ± 10.68	44.21 ± 9.93	28.91 ± 5.93
	Trans-ferulic acid	93.46 ± 14.94 ab	116.46 ± 33.56 a	39.65 ± 9.27 b
	Cis-ferulic acid	14.18 ± 2.77	23.12 ± 5.53	12.32 ± 1.58
Flavanols (mg/L)	(+)-Catechin	305.69 ± 75.79 a	35.45 ± 4.13 b	nd
	(-)-Epicatechin	41.19 ± 7.42	33.75 ± 5.00	nd
Flavonols (mg/L)	Rutine	48.00 ± 9.77 a	34.04 ± 8.13 ab	9.40 ± 2.73 b
	Myricetin-3-*O*-galactoside	38.16 ± 6.75	48.92 ± 9.54	23.82 ± 12.24
	Quercetin-3-*O*-galactoside	19.99 ± 6.23 b	45.66 ± 7.50 a	24.64 ± 6.41 ab
	Quercetin-3-*O*-glucoside	11.42 ± 3.94 b	39.31 ± 11.32 a	22.95 ± 6.41 ab
	Kaempferol-3-*O*-glucoside	nd	11.82 ± 3.11	nd
	Kaempferol	23.25 ± 3.76 a	11.28 ± 2.58 b	11.20 ± 4.14 b

Values are expressed as mean ± standard error. Different lowercase letters indicate differences according to ANOVA and LSD test ($p < 0.05$).
